# Antibacterial and antiplaque efficacy of a lactoperoxidase-thiocyanate-hydrogen-peroxide-system-containing lozenge

**DOI:** 10.1186/s12866-021-02333-9

**Published:** 2021-11-03

**Authors:** A. Welk, S. Patjek, M. Gärtner, R. Baguhl, Ch. Schwahn, H. Below

**Affiliations:** 1 Department of Restorative Dentistry, Periodontology, Endodontology, Preventive and Pediatric Dentistry, Dental School of the University Medicine Greifswald, Walther-Rathenau-Str. 42a, 17475 Greifswald, Germany; 2 Institute of Hygiene and Environmental Medicine of the University Medicine Greifswald, Greifswald, Germany; 3 Dental School, Department of Prosthodontics, University of Greifswald, Greifswald, Germany

**Keywords:** Mouth hygiene product, Antiseptic, Lactoperoxidase-thiocyanate-hydrogen peroxide-system-containing lozenge, Essential oil, Dental plaque

## Abstract

**Background:**

Antimicrobial agents are considered valuable adjuncts to mechanical methods of plaque control. However, their long-term use can be limited because of side effects.

Therefore, using physiological substances is promising due to no risk of development, for example, of microbial resistances, allergies or DNA damaging. The lactoperoxidase-thiocyanate-hydrogen peroxide system (LPO-system) is a highly effective antimicrobial system. This study aimed to evaluate in a randomized study with a four-replicate cross-over design the effectiveness of two oral hygiene lozenges containing LPO-system in oral hygiene.

**Results:**

After using the mouth rinse as positive control (A) and allocated test lozenges (B) (0.083% H_2_O_2_) & (C) (0.04% H_2_O_2_) for 4 days instead of the normal oral hygiene procedures (tooth brushing etc.), Listerine rinse (A) was statistically significantly more effective than the LPO-system-lozenge with 0.083% H_2_O_2_, the LPO-system-lozenge with 0.04% H_2_O_2_, and the placebo lozenge (D) in inhibiting plaque. Lozenges B and C were statistically significantly more effective than the placebo lozenge, but no statistically significant differences could be observed between them.

The LPO-system-lozenge (B) reduced statistically significantly more *S. mutans* than the LPO-system-lozenge with (C) and the placebo lozenge (D). The LPO-system-lozenge (C) reduced statistically significantly more *Lactobacilli* than Listerine (A), the LPO-system-lozenge (B) and the placebo lozenge (D). There were no statistically significant differences in the total CFUs between Listerine rinse, the LPO-system-lozenge with 0.083% H_2_O_2_ (B), the LPO-system-lozenge with 0.04% H_2_O_2_ (C), and the placebo lozenge (D). On day 5 there were no differences of the OSCN^−^-values between all A, B, C, and D. However, the SCN^−^-values increased over the days in both LPO-system-lozenges (B/C). The statistically significant differences between B/C and A/D on day 5 were as followed: A to B *p* = 0.0268; A to C *p* = 0.0035; B to D *p* = 0.0051; C to D *p* = 0.0007. Only in the group of Listerine (A) increased the NO_3_^−^/NO_2_^−^-quotient over the test time, which indicates a reduction of nitrate-reducing bacteria. On Day 5 the statistically significant difference between A and B was *p* = 0.0123.

**Conclusions:**

The results indicate that lozenges containing a complete LPO-system, inhibiting plaque regrowth and reducing cariogenic bacteria, may be used in the daily oral hygiene.

## Background

Antimicrobial agents are considered valuable adjuncts to mechanical methods of plaque control, especially in cases of insufficient oral hygiene such as during orthodontic multibracket therapy. However, their long-term use can be limited because of local side effects and even for microbiostatic active agents, the risk of developing resistance or cross-resistance against antibiotics [1]. Therefore, using physiological substances in the sense of supporting the body’s self-defense system is promising due to no risk of development, for example, of microbial resistances, allergies or DNA damaging. The lactoperoxidase-thiocyanate-hydrogen peroxide-system (LPO-system) existing among other peroxidase systems in saliva is a highly effective antimicrobial system, which based on the lactoperoxidase-mediated oxidation of thiocyanate to the active agent hypothiocyanite [2].

The LPO-system has in addition to bacteriostatic or bactericidal effects also antiviral effects. Further, it inactivates carcinogenic and mutagenic substances and prevents the accumulation of cytotoxic hydrogen peroxide [3, 4]. Thus, it is also protective for periodontal structures as an antioxidant reducing the oxidative stress associated with negative inflammatory responses and damaging directly periodontal ligament cells by inducing their apoptosis [5].

LPO, thiocyanate (SCN^−^) and H_2_O_2_ are natural components of the human saliva. Salivary glands and the activated neutrophils of the saliva and the gingival sulcus are the endogenous sources of H_2_O_2_ and SCN^−^ [6]. H_2_O_2_ is also formed by a series of oral microorganisms [7] and is considered a critical factor to maintain microbial symbiosis.

After discovering the effectiveness of oral peroxidase’s antimicrobial function in the oral cavity, some companies have tried to use this natural system in dentifrices, mouth rinses, moisturizing gels or mouth sprays [4, 8, 9].

However, the in vivo studies on such products haven’t shown reliable effectiveness at all compared with the convincing results of the enzyme system’s antimicrobial effectiveness in vitro studies [10].

Some authors could show positive effects regarding reducing the salivary levels of cariogenic bacteria, plaque accumulation and prevention of gingivitis, and dental caries [4, 11–15]. The results in the study of Midda 1986 [16], however, showed no difference in plaque scores but a significant reduction in gingivitis scores in the enzyme-containing dentifrice group. Maybe the reason for that is a positive influence of the enzyme-containing dentifrice on the microbiome more associated with health. Adams et al. 2017 determined the effect of a toothpaste containing enzymes (e.g. LPO-system) and proteins on plaque oral microbiome ecology by using DNA sequencing. The used toothpaste led to a positive shift to a microbiome more associated with health. In comparison to a toothpaste without enzymes and proteins, it significantly increased the relative abundance of health-associated organisms in plaque whilst driving a concomitant decrease in a number of disease-associated organisms over time [17].

Other authors found no antibacterial effects, reductions in plaque formation or acidogenicity in subjects using enzyme-containing dentifrice [8, 9].

However, either the generated HOSCN/OSCN^−^ level was not measured or the observed raised level led to no bactericidal effect [8, 9].

The reasons for that are seen in the following issues:

- missing of components (hydrogen peroxide is seen as the limiting factor in the literature [8, 18], however, we could show that not only the increase of hydrogen peroxide or/and thiocyanate is important to get a sufficient antimicrobial effect but also the raise of LPO [19, 20];

- quality of LPO (a stabilized LPO with high activity is expensive and is not used in toothpastes);

- interaction between the ingredients (keeping the quality of all three components within one toothpaste by avoiding reactions between the components in the toothpaste, especially in an aqueous environment, is difficult) [21];

- relatively low substantivity [8];

- complexity of the lactoperoxidase-thiocyanate-hydrogen peroxide system and its sensibility on environment changes (e.g. pH values, temperature) [21].

Morita et al. 2017 [22] and NAKANO et al. 2019 [23] considering already some of these aspects, could show positive effects of tablets containing lactoferrin and lactoperoxidase regarding reduction of periodontitis strains and gingival inflammation.

Both used glucose oxidase (GO) to provide the LPO-system with hydration peroxide.

To be independent of the unpredictable hydration peroxide generation via GO and to see the pure effect of LPO-system-based lozenges, we tested lozenges containing all three components of the LPO-system with two different H_2_O_2_ concentrations (0.083%/0.04%) in a crossover study.

## Results

### Plaque re-growth study

**Fig. 1:**
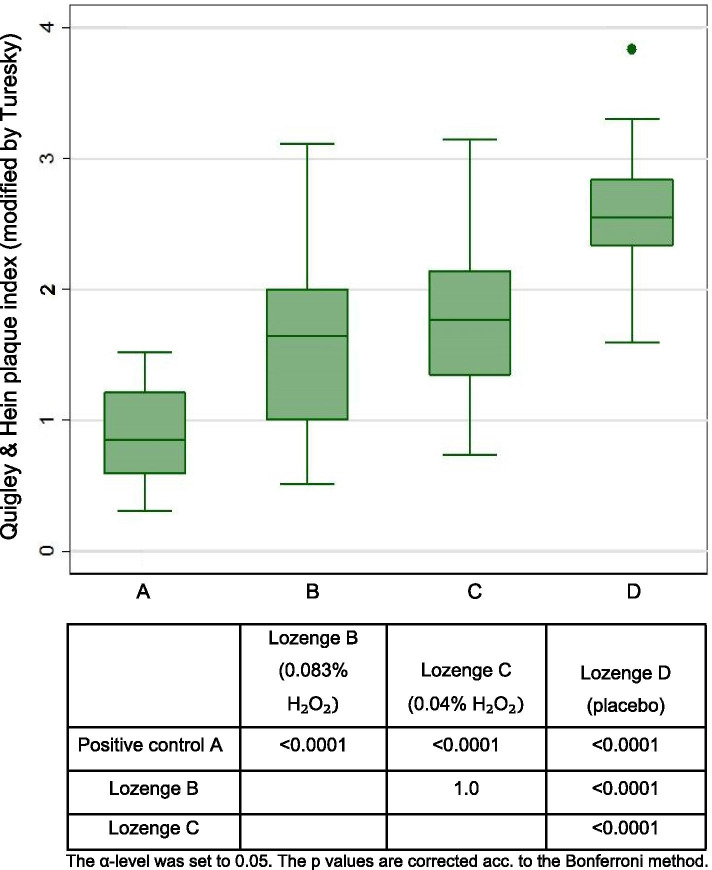
Four-day plaque regrowth study: Box plot for plaque regrowth after 4 days (*n* = 16 in each group, because of the cross-over design)

The plaque index data for each treatment are shown in Fig. 1. Comparisons of pairs of treatments indicated that after 4 days, Listerine rinse was statistically significantly more effective in inhibiting plaque (median QHI 0.88) than the LPO-system-lozenge with 0.083% H_2_O_2_, (median QHI 1.6), the LPO-system-lozenge with 0.04% H_2_O_2_, (median QHI 1.8), and the placebo lozenge (median QHI 2.6). Lozenges (0.083% H_2_O_2_) and (0.04% H_2_O_2_) were statistically significantly more effective than the placebo lozenge, from the ordinal regression, the odds ratios (OR) were 0.0043 (95% CI: 0.0007–0.0254) and 0.0137 (95% CI: 0.0027–0.0688), respectively. An OR can be converted into the number needed to treat (NNT) assuming a certain risk in unexposed subjects (r_0_) using the formula NNT = (r_0_ * OR – r_0_ + 1)/(r_0_*(r_0_–1)*(OR – 1)) (Doi et al.). Thus, assuming r_0_ = 0.1 (10%), both NNT are 10; assuming r_0_ = 0.5 (50%), both NNT are 2. No statistically significant difference could be observed between the lozenges (0.083% H_2_O_2_) and (0.04% H_2_O_2_) (Fig. 1).

### Bacterial count measurements

**Fig. 2:**
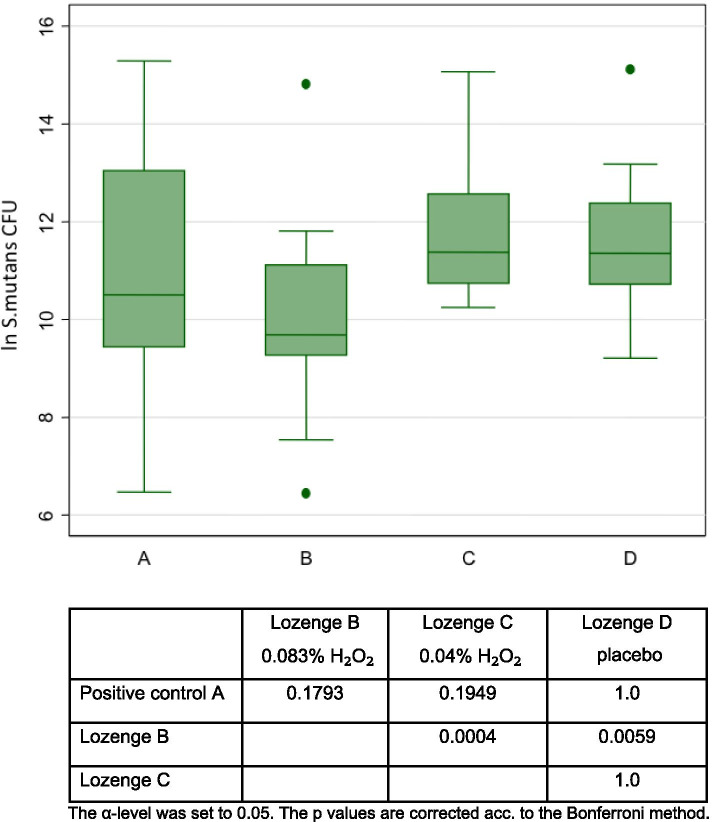
Boxplot showing bacterial counts of *S. mutans* (colony forming units per sample, log transformed) on the tooth surface for 4 treatments (n = 16)

**Fig. 3:**
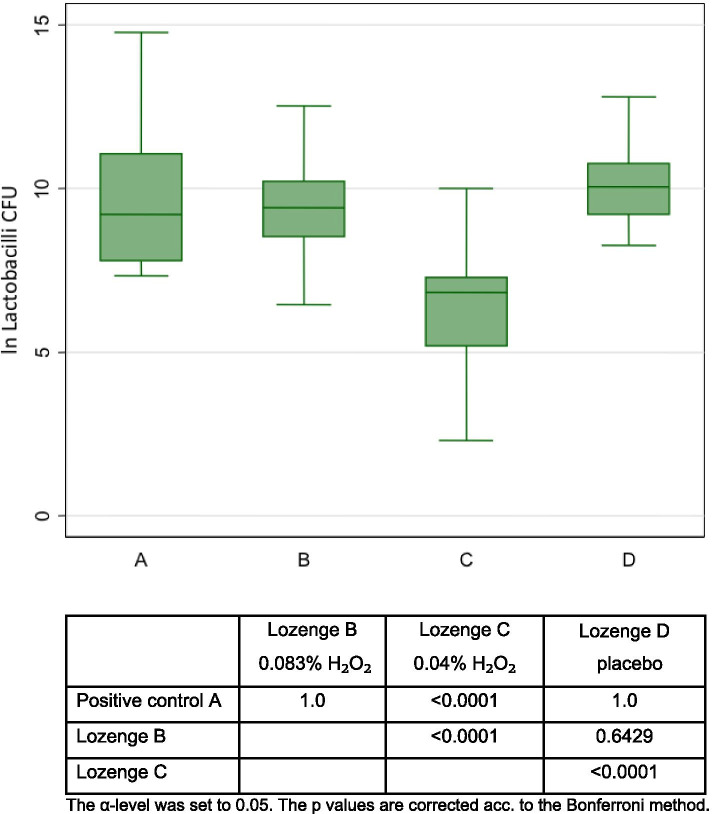
Boxplot showing bacterial counts of *Lactobacilli* (colony forming units per sample, log transformed) on the tooth surface for 4 treatments (n = 16)

**Fig. 4:**
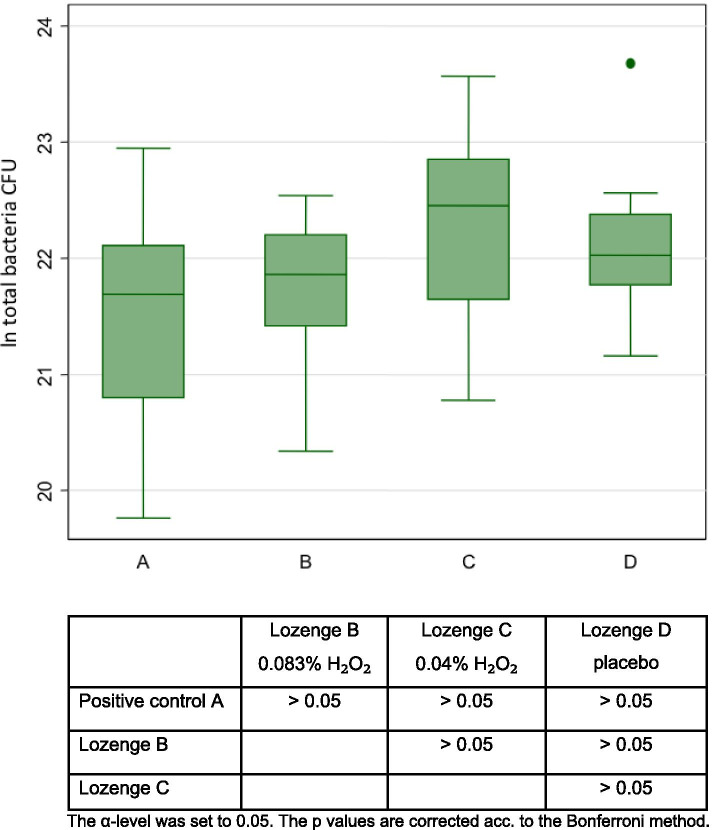
Boxplot showing total bacterial count (colony forming units per sample, log transformed) on the tooth surface for 4 treatments (n = 16)

The results of bacterial count measurements of plaque samples for each treatment are presented in Figs. 2, 3 and 4.

### *S. mutans*

The LPO-system-lozenge with 0.083% H_2_O_2_ reduced statistically significantly more *S. mutans* than the LPO-system-lozenge with 0.04% H_2_O_2_ and the placebo lozenge (Fig. 2). There was no difference between Lozenges (0.083% H_2_O_2_) and Listerine^®^.

### Lactobacilli

The LPO-system-lozenge with 0.04% H_2_O_2_ reduced statistically significantly more *Lactobacilli* than Listerine^®^, the LPO-system-lozenge with 0.083% H_2_O_2_ and the placebo lozenge (Fig. 3).

### Total bacterial count

There were no statistically significant differences in the total CFUs between Listerine rinse, the LPO-system-lozenge with 0.083% H_2_O_2_, the LPO-system-lozenge with 0.04% H_2_O_2_, and the placebo lozenge (Fig. 4).

### LPO system- and bacteria-related analytical measurements

The values are shown in Table 1. On day 5 there were no differences of the OSCN^−^-values between all groups. However, the SCN^−^-values increased over the days in both LPO-system-lozenge groups. The statistically significant differences between Lozenge (0.083% H_2_O_2_) / Lozenge (0.04% H_2_O_2_) and Listerine^®^ / placebo lozenge on day 5 were as followed: Listerine^®^ to Lozenge (0.083% H_2_O_2_) *p* = 0.0268; Listerine^®^ to Lozenge (0.04% H_2_O_2_) *p* = 0.0035; Lozenge (0.083% H_2_O_2_) to placebo lozenge *p* = 0.0051; Lozenge (0.04% H_2_O_2_) to placebo lozenge *p* = 0.0007. Only in the Listerine-group increased the NO_3_^−^/NO_2_^−^-quotient over the test time, which indicates a reduction of nitrate-reducing bacteria. On Day 5 the statistically significant difference between Listerine^®^ and Lozenge (0.083% H_2_O_2_) was *p* = 0.0123.

**Table 1:** LPO-system- and bacteria-related analytical parameters on *day one* before professional tooth cleaning, on *day three* before, and on *day five* one hour after using allocated lozenges or mouth rinse

	OSCN^−^ (mg/l)			SCN^−^ (mg/l)			NO_3_^−^/NO_2_^−^		
	Day 1	Day 3	Day 5	Day 1	Day 3	Day 5	Day 1	Day 3	Day 5
	Mean ± SD	Mean ± SD	Mean ± SD	Mean ± SD	Mean ± SD	Mean ± SD	Mean ± SD	Mean ± SD	Mean ± SD
A	0.62 ± 0.37	0.52 ± 0.47	0.47 ± 0.38	92.34 ± 48.51	114.96 ± 61.52	113.76 ± 62.50^(1.2)^	3.10 ± 2.31	6.00 ± 3.99	5.91 ± 4.42^(5)^
B	0.64 ± 0.41	0.49 ± 0.31	0.62 ± 0.32	88.36 ± 47.03	127.02 ± 43.30	154.98 ± 68.61^(1.3)^	3.84 ± 2.25	2.74 ± 1.19	2.66 ± 1.97^(5)^
C	0.81 ± 0.78	0.64 ± 0.38	0.55 ± 0.49	120.13 ± 82.01	156.73 ± 68.86	164.63 ± 60.71^(2.4)^	2.67 ± 2.72	3.17 ± 2.44	3.54 ± 2.72
D	0.50 ± 0.28	0.53 ± 0.46	0.58 ± 0.39	103.03 ± 61.27	148.55 ± 102.59	103.48 ± 50.18^(3.4)^	2.44 ± 1.66	3.57 ± 2.07	3.46 ± 2.96

The α-level was set to 0.05. The *p* values are corrected acc. to the Bonferroni method

1) A to B *p* = 0.0268; 2) A to C *p* = 0.0035; 3) B to D *p* = 0.0051; 4) C to D *p* = 0.0007; 5) A to B *p* = 0.0123

## Discussion

The tested LPO-system-lozenges with 0.083% H_2_O_2_ and with 0.04% H_2_O_2_ reduced statistically significantly more plaque, *S. mutans*, and *Lactobacilli* than the placebo but not total bacterial count.

The used 4-day plaque regrowth study with its double-blinded, placebo-controlled, randomized, four-replicate cross-over design is seen as very sufficient in the initial clinical test phase of new antimicrobial agents or new dental hygiene products.

We differ from the double-blinded standard regarding the positive control due to compare the lozenges not just to a negative control (placebo) but also to an oral hygiene benchmark product and common oral hygiene procedure, respectively. Therefore, on one hand, there were no differences between the test and placebo lozenges in shape, color, and smell. But on the other hand, we used the mouth rinse Listerine^®^ Total care™ (Johnson & Johnson GmbH, Germany) as positive control. However, this discrepancy had no influence on the QHI measuring, because the calibrated examiner (ICC > 70) did not know what the participant used.

Lenander-Lumikari, M. et al. 1995 evaluated the Salivette® kit used to take saliva samples [24]. We used cortisol salivettes (c-salivettes) only for the determination of anions in saliva samples by ion chromatography. In a pilot study, we validated the method by determination of recovery rates [25]. Thus, the used c-salivettes are sufficient for our study goal to measure the saliva parameter at different time points.

A wash-out period of 10 days is seen as sufficient to avoid carry-over effects [26]. However, in the interpretation of the results, it should be considered that this depends also on the used agents.

The results of our study are in line with other studies showing a reducing effect on plaque and caries bacteria by hygiene products containing components of the LPO-systems [4, 11–14, 16].

However, there are some studies with no effects [8, 9, 27].

One reason for the contrary results could be the difficulties in containing active enzymes in an aqueous environment, such as toothpastes, so that they do not degrade during long-term storage at room temperature. For example, Nimatullah et al. 2011 showed that LPO stored in the aqueous environment quickly loses its activity. They demonstrated a total loss of its activity at 25 °C during the first week [21]. Further, Lenander-Lumikari et al. 1993 fund in their brushing experiments with an LPO-system-containing toothpaste (Biotene^®^) a relative high range of the generated HOSCN/OSCN^−^ level among the subjects (from 95 to 300 μM) [8]. It seems that the subjects themselves play a decisive role in generating HOSCN/OSCN^−^ [8].

As already mentioned, the limiting factors for the optimal generation of hypothiocyanite ion is seen in the low level of hydrogen peroxide [18]. However, adding H_2_O_2_ to a dental hygiene product is restricted to 0.1% by the European Scientific Committee on Consumer Products. Independently of this, it is difficult to keep the LPO-system stable in an aqueous environment, if all three components are included. This applies particularly to pure hydrogen peroxide. Therefore, some LPO-based products contain amyloglucosidase (AMG) and GO [4, 28] or glucose/GO [22, 23] trying to generate H_2_O_2_.

In the study of Cawley et al. 2019, the concentration of hydrogen peroxide in saliva was 64% greater after brushing with the toothpaste containing enzymes (AMG/GO) (4.2 μM) than after brushing with the control toothpaste without enzymes (2.7 μM). However, the authors used an analytical method being non-specific to H_2_O_2_ and detecting all peroxides [28].

On the other hand, Patel et al. 2006 showed in their study that a concentration level of H_2_O_2_ over 40 mM impairs the LPO enzyme [29].

However, Fonteh et al. (2005) found an optimal LPO activity of about 100 μmol/l. At higher concentration, the OSCN^−^ producing activity of LPO is inhibited [30]. Thus, it seems that the optimum OSCN^−^ production is between 20 and 100 μmol/l H_2_O_2_ [31].

Consequently, the range of the necessary H_2_O_2_ to get the optimal HOSCN/OSCN^−^ generation is relatively small.

We used carbamide peroxide, because the generation of a sufficient amount of hydrogen peroxide by an enzyme cascade is time-delayed and not always ensured.

Overall, the right concentration levels of LPO enzyme, thiocyanate and hydrogen peroxide to each other are very important to get the biggest yield of OSCN^−^ in the oral cavity. Especially the ratio of SCN^−^ to H_2_O_2_ plays an important role in the optimal formation of OSCN^−^. While Mansson-Rahemtulla et al. 1983 suggested to use H_2_O_2_ in excess over SCN^−^, today, the opposite is believed – using SCN^−^ in excess over H_2_O_2_ due to avoid overoxidation [32].

Our lozenges contained SCN^−^ to H_2_O_2_ in proportion of 2 to 1 (B) and 4 to 1 (C, by half of the H_2_O_2_ content in B) assuming that under optimal conditions all H_2_O_2_ should have been consumed and converted into OSCN^−^. In addition of achieving a high active antimicrobial compound, the complete conversion of H_2_O_2_ into OSCN^−^ is the prerequisite for avoiding oxidative stress through the added carbamide peroxide.

We used carbamide peroxide as fast bioavailable H_2_O_2_ source, which is converted immediately by LPO and other oral enzymes. Further we used LPO in a high concentration. Thus, we assume to avoid oxidative stress.

Tenovuo et al. 1981 could achieved more effective inhibition of plaque acid production by increasing the concentration of OSCN^−^ ions by only supplementing H_2_O_2_ and SCN^−^ in final concentrations of 700 μM and 10 mM, respectively [33].

Lenander-Lumikari et al. 1992 achieved a complete loss of viability of *Candida albicans* with 0.2 mM KSCN and 300 μM H_2_O_2_, though the HOSCN/OSCN^−^ concentrations did not exceed 100 μM. The effect was accomplished only without phosphate due to the physiological saliva concentration of phosphate blocked the antifungal effect of the peroxidase systems [34]. In opposite to that, Welk et al. 2009 showed in their saliva suspension tests that increasing only the level of SCN^−^ and hydrogen peroxide over the physiological level was not clinically relevant regarding antibacterial or antifungal effectiveness. However, increasing all three components, incl. lactoperoxidase enzyme, over their physiological level was very effective in bacteria and *candida albicans* reduction [19]. This is in line with the observations of Tenovuo & Knuuttila 1977 that the LPO activity should be approximately 2 to 3 times higher (10 U/ml) than the LPO activity normally found in human whole saliva (3 to 5 U/ml) [35].

The tested lozenges contained all three components of the LPO-systems including H_2_O_2_-generating carbamide peroxide, equivalent to 0.083 and 0.04% H_2_O_2_. Dry lozenges have the advantage of keeping the LPO activity and carbamide peroxide more stabilized than in toothpastes with their aqueous environment.

The LPO-system-lozenge with 0.083% H_2_O_2_ (B) and the LPO-system-lozenge with 0.04% H_2_O_2_ (C) reduced *S. mutans* & *Lactobacilli* statistically significantly more than placebo (D) and (C) even more than Listerine^®^ (A). However, there were no statistically significant differences in the total CFUs between A, B, C, and D. Thus, the LPO-system-lozenges reduce specifically cariogenic bacteria such as *S. mutans* and *Lactobacilli*, which is in line with other studies [4, 36] .

These different sensitivities of the bacteria are the result of the different cell wall structures and the presence of different defensive barriers [37]. OSCN^−^ is antibacterial in different ways. It reacts with SH-groups of essential enzymes or other proteins with sulfenyl groups [10, 38] inhibiting glycolysis [39, 40]. Further, the structural damage to the microbial cytoplasmic membrane by the oxidation of SH-groups leads to a loss of Na^+^, amino acids, and peptides. The uptake of glucose, amino acids, purines and pyrimidines in the cell and the nucleic acid and protein synthesis are then also inhibited [41].

The ability to recover from the inhibition depends on the NAD(P)H - OSCNˉ oxidoreductase system of the species [42, 43].

The higher concentration level of hydrogen peroxide (0.083%) of the lozenge B was more effective in inhibiting *S. mutans* than the lozenge C with 0.04% H_2_O_2_. However, the lower concentration level of hydrogen peroxide (0.04%) of the lozenge C was more effective in inhibiting *Lactobacilli* than the lozenge B with 0.083% H_2_O_2_. The reasons for these controversy results can’t be said exactly at moment because these are initial concentrations in the lozenges and we did not measure H_2_O_2_ in the saliva.

Even though we assume that all H_2_O_2_ was consumed totally for the OSCN-production, we can’t be sure that this really happened. Therefore, H_2_O_2_ could have a local and/or for a short time a direct effect on the oral bacteria inhibiting *S. mutans* more than *Lactobacilli*, because *S. mutans* is more sensitive to H_2_O_2_ than H_2_O_2_-generating bacteria such as *Lactobacilli* [44]. Further, *Lactobacilli* can also have strains, which can and cannot produce H_2_O_2_, respectively [45]. However, our bacterial detection test does not differentiate into different strains of *S. mutans* and *Lactobacilli.* Thus, we have no overview of the portion of H_2_O_2_-generating and H_2_O_2_ non-generating of *Lactobacilli*. Further studies are necessary to clarify these observed findings.

Despite the inhibition of evaluated cariogenic bacteria, the total CFUs were not statistically significantly reduced. Thus, the LPO-system-based lozenges had no effect on the total bacterial count but a positive effect on the bacterial composition of the oral biofilm regarding caries-related bacteria without disturbing the commensal bacteria. This is in the line with the study of Adams et al. 2017 determining the effect of a toothpaste containing enzymes (e.g. LPO-system) and proteins on plaque oral microbiome ecology by using DNA sequencing [17]. The used toothpaste led to a positive shift to a microbiome more associated with health, significantly increasing the relative abundance of health-associated organisms in plaque whilst driving a concomitant decrease in several disease-associated organisms compared with a toothpaste without enzymes and proteins over time.

This would be exactly what we are looking for: no reducing all bacteria very effectively but only the pathogens and giving a favor to the commensal flora. It seems that saliva enzymes have this potential to do that [17]. However, the substantivity of OSCNˉ/HOSCN is in comparison to common antimicrobial agents low [8, 32]. This is also reflected in our OSCN^−^ values. There were no statistically significant differences between A, B, C and D at all three measurement times. Thus, our data confirmed the results of other studies. Mansson-Rahemtulla et al. (1983), for example, got only directly after 2 min rinsing with a mouth rinse containing all three components of the LPO-system a clinically relevant level of > 100 μM OSCNˉ/HOSCN. Already 1 min after mouth rinsing, the OSCNˉ/HOSCN value dropped from 135 ± 30 μM to 58 ± 18 μM and after 5 min to 13 ± 20 μM OSCN^−^ [32]. In the brushing experiments of Lenander-Lumikari et al. 1993 with a LPO-system-containing toothpaste (Biotene^®^), the generated OSCNˉ/HOSCN levels returned to the baseline salivary levels already in 20 min [8].

The decomposition of OSCNˉ/HOSCN can be spontaneous or induced by thiols [46].

The lozenges have the advantages of a retarded release of all LPO-components over the whole dissolving time producing continually OSCNˉ/HOSCN. The dissolving time of our lozenges lasted approximately 10–15 min. The dissolving time can be adjusted by the hardness of the lozenges.

Based on our lifestyle, several food impulses over the day are common. Therefore, we let our subjects suck the lozenges five times a day. Three times after meals and two times between them.

Despite intaking of all three components over four days only SCN^−^ increased over this period, which based on the test-lozenges. On day 5 the statistically significant differences between A, D and B, C were around one third. Thus, the differences lay below the differences between smoker and non-smoker, which is the two- until four-fold [37]. Independently on this, Chandler & Brian 2015 consider the moderate increase SCN^−^ not only as harmless but they reported in their review that an increased SCN^−^ plasma level may be a protective factor of cardiovascular diseases [47].

The variations between the three measurement days in group A and D can be considered as nutrition-related. The intake of cyanide- and thiocyanate-containing foods like broccoli, cauliflower or beans, and peas, for example, increases the SCN^−^ level in blood, saliva, and tissue [41].

The NO_3_^−^/NO_2_^−^ level between Listerine^®^ and lozenges A was statistically significantly different on day 5. Already after three days, the NO_3_^−^/NO_2_^−^ value doubled in the Listerine^®^ group. This is in line with the experiments of Petersson et al. 2009, in which the used antiseptic mouthwash (0.2% CHX containing Corsodyl Mouthwash, GlaxoSmithKline, Brentford, England) reduced also the nitrate-reducing bacteria significantly [48]. However, our LPO-system-based lozenges reduced cariogenic bacteria, such as *S. mutans* and *Lactobacilli*, without diminishing nitrate-reducing bacteria, which reduce NO_3_ˉ to NO via NO_2_ˉ [49]. Doel et al. (2004) suspect a cardio-protective effect by the presence of NO2ˉ or NO3ˉ and nitrate-reducing bacteria. The positive influence of NO on the organism, in general, has been well documented [50]. Although the exact mechanism behind the lowering of blood pressure by the uptake of NO_3_ˉ and NO_2_ˉ is still not fully understood, it can be summarized that the symbiotic oral bacteria play an active role in the regulation of the gastrointestinal tract as well as the cardiovascular system [48]. On the other hand, in the presence of nitrite, the formation of carcinogenic nitrosamines is possible. In this case, the reduction of nitrite could be an advantage [51]. Therefore, a plaque-inhibiting dental care product that does not inhibit nitrate-reducing bacteria would be beneficial.

Overall, using saliva components instead of conventional antiseptics to keep the symbiosis or to remodel dysbiotic communities back to a state of symbiosis with the host is interesting. It would be a further step of taking into account ecological aspects in a modern approach in the prevention of oral diseases [52].

In future studies, it should be tested whether optimized LPO-system-based lozenges in combination with additional agents (such as lysozyme, lactoferrin and fluorides or stimuli of the enzymatic activity by natural products such as 6-gingerol [53]) are able to improve the showed results.

## Conclusion

The results indicate that lozenges containing a complete-LPO-system, inhibiting plaque regrowth and reducing cariogenic bacteria effectively, may be used as an in-between oral hygiene product.

## Materials and methods

The 4-day plaque regrowth study employed a double-blind (regarding lozenges), placebo-controlled, randomized, four-replicate cross-over design, described by Addy et al. 1983, in which each subject served as its own control [54].

The study design is very common and widespread for the evaluation of the efficiency of antimicrobial substances in oral cavity. Clinical study procedures were performed according to the ethical standards of the national research committee and the declaration of Helsinki 1964. Approval for all clinical procedures and the trial was obtained by the ethical committee of the University of Greifswald (Code BB 015/14). The clinical trial was registered in the German Database for clinical trials (DRKS00022810, date of registry: 02.09.2020).

Sixteen healthy volunteers (dental students of the University of Greifswald) were selected who had at least 24 caries-free teeth with inflammation free gingiva. Exclusion criteria were smoking, concurrent participation in another clinical trial, taking antibiotics. There were no dropouts.

The written informed consent was obtained from every participant to any study-related procedures. As an efficient sample size estimation requires an estimate of a correlation coefficient, which is usually not available [55], we used a simple approach to determine the sample size and looked at the sample size of successful crossover trials with a similar research question as a good indicator for the sample size needed [56]. Thus, based on literature such as Moran et al. 1994 [57] or Rosin et al. 2002 [58], the sample size of 16 participants could be considered as appropriate for our highly efficient study design [55].

All subjects were randomly assigned a number by H.B., which determined the order of application of the following lozenges or mouth rinse: sequence_1_ = A,B,C,D (3); sequence_2_ = B,C,D,A (4); sequence_3_ = C,D,A,B (3); sequence_4_ = D,B,A,C (5); and sequence_5_ = D,A,B,C (1). The subjects and the examiner (M.G.) were blinded except for treatment A.

The positive control (A) was a commercially available essential oil mouth rinse (Listerine^®^ Total care™, Johnson & Johnson GmbH, Germany).

The tested oral hygiene lozenges (B/C) were sugar alcohol-based (xylitol, sorbitol, mannitol) lozenges containing the complete LPO-system (10 mg LPO 350 U/mg (Sternenzym, Germany), 7,5 mg KSCN) with.

- 0.083% H_2_O_2_ (provided by carbamide peroxide) accordingly a 1:2 H_2_O_2_/SCN^−^ relation (Lozenge - B),

- 0.040% H_2_O_2_ (provided by carbamide peroxide) accordingly a 1:4 H_2_O_2_/SCN^−^ relation (Lozenge - C).

The negative control (D) was a sugar alcohol-based (xylitol, sorbitol, mannitol) placebo lozenge without LPO-system components.

### Clinical trial

On day 1 of each study period an intraoral examination of the teeth and soft tissue was followed by a professional tooth cleaning. After instruction, the volunteers got their allocated lozenges or mouth rinse. Instead of the normal oral hygiene procedures (tooth brushing etc.), the subjects sucked 5 times per day (08:00 AM/ after breakfast, 11:00 AM, 02:00 PM, 05:00 PM and 08:00 PM/after last meal) the allocated lozenges (10–15 min) or rinsed twice per day (08:00 AM/after breakfast, 08:00 PM/after last meal) the mouth rinse (20 ml, 1 min rinse) for the next 4 days.

The last use of the lozenges or the mouth rinse took place 1 hour before the evaluation on day 5.

On this day, the plaque regrowth was assessed with a disclosing solution (MIRA-2-TON^®^, Hager & Werken GmbH, Germany) and scored using Quigley & Hein plaque index (QHI) modified by Turesky et al. 1970 [59].

### Plaque sampling and microbiological evaluation procedure

After that, the plaque samples were taken from the buccal surfaces of the first and third quadrant, and the palatinal/lingual surfaces of the second and fourth quadrant, respectively. All samples of each subject were pooled before processing for the microbiological analysis.

The pooled plaque sample was transferred to a vial. The plaque was determined by weighing and suspend in 0.9% NaCl. Fifty microliter of the plaque suspension were pipetted onto the agars in a 1:10 dilution. After 48 h of incubation at 37 °C, the colonies were counted. The total bacterial count was determined on the basis of the German Industry Standard (DIN EN ISO 6222) on tryptone soy agar after 48–72 h incubation at 36 °C [60]. The isolation and differentiation of the pathogens *S. mutans* and *Lactobacilli* took place quantitatively with common agars (Ivoclar-Vivadent, Lichtenstein) for this purpose. The *S. mutans* was detected with a Mitis Salivarius agar, which contains bacitracin [61] and the *Lactobacilli* with the Rogosa agar [62].

The antibacterial effect was assessed by the determination of colony-forming units (CFU; *S. mutans, Lactobacilli* and total bacterial count).

### Saliva sampling and analytical evaluation procedure

Saliva samples for ion chromatography were collected by polypropylene Cortisol Salivettes^®^ (Sarstedt, Nümbrecht, Germany). The synthetic swab was moved by the tongue in the mouth for 1 min with a regular frequency to absorb saliva sufficiently.

After that, the swab was returned into labeled c-salivette and stored on ice for a short moment until the c-salivette was centrifuged at 1000×g for 2 min yielding a clear saliva sample. These samples were immediately placed on ice and analyzed within 30 min at the laboratory of the Institute of Hygiene and Environmental Medicine, Greifswald.

The analytic of the LPO system parameters (OSCN^−^, SCN^−^) and bacteria-related parameters (nitrite (NO_2_^−^) and nitrate (NO_3_^−^)) was done on day 1 before professional tooth cleaning, on day three before and on day five 1 hour after using allocated lozenges or mouth rinse by validated ion chromatography method of Below et al. 2018 using a Professional IC 850 equipped with an amperometric detector, a conductive detector and a scanning UV detector (Deutsche METROHM, Filderstadt, Germany) [25].

Each test cycle was followed by at least 10 days wash-out period. In this time the subjects resumed their normal oral hygiene procedures with tooth brush and tooth paste for 7 days and suspended normal oral hygiene procedures for the last 3 days to support the recovery of the oral microbiome.

Data were analyzed using procedure pkcross in Stata (StataCorp LP/version 14.2, College Station, TX, USA), which was tailored to analyze a cross-over design in a linear regression model. In special terms of the procedure pkcross, variables for treatment, period, sequence, and the id were included; a potential carryover effect was not modeled [63]. To get the OR, which is superior to the relative risk for calculating the NNT [64], we used the ordinal logistic regression model with a parameterization corresponding to the procedure pkcross. This OR can be interpreted as for a binary endpoint, whatever the cutoff of the ordinal endpoint is.

For all analyses the α-level was set to 0.05. The *p* values are corrected acc. to the Bonferroni method for multiple comparisons.

## Data Availability

All data generated or analyzed during this study are included in this published article.

## Abbreviations

AMG: Amyloglucosidase
CFU: Colony-forming units
DNA: Deoxyribonucleic acid
GO: Glucose oxidase
LPO: Lactoperoxidase
NAD(P)H: Nicotinamide adenine dinucleotide phosphate
*S. mutans*: *Streptococcus mutans*
